# Supplementary material for: Empirical methods for controlling false positives and estimating confidence in ChIP-Seq peaks
Source: BMC Bioinformatics. 2008 Dec 5;9:523. doi: 10.1186/1471-2105-9-523 (PMC2628906; doi:10.1186/1471-2105-9-523)
Supplement: Additional File 1 — A variety of html documents from the USeq web site detailing the available applications, their best usage, output file type descriptions, command line menus, etc. [file 1471-2105-9-523-S1.zip › USeqUserGuides/JavaDoc.zip!/JavaDoc/util/gen/Passwords.html]

Passwords


|  |  |  |
| --- | --- | --- |
| **Overview** \| **Package** \| **Class** \| **Use** \| **Tree** \| **Deprecated** \| **Index** \| **Help** | |  |
| **PREV CLASS**   **NEXT CLASS** | **FRAMES**    **NO FRAMES**     **All Classes** |  |
| SUMMARY: NESTED \| FIELD \| CONSTR \| METHOD | DETAIL: FIELD \| CONSTR \| METHOD |  |


---


## util.gen Class Passwords

```
java.lang.Object
  util.gen.Passwords
```

---

``` public class Passwords extends java.lang.Object ```

Methods for generating one time passwords.

**Author:**
:   nix

---

| **Field Summary** | |
| --- | --- |
| `static java.lang.String[]` | `nonAmbiguousLetters` |
| `static java.lang.String[]` | `numberLetters` |


**Constructor Summary**

`Passwords()`


| **Method Summary** | |
| --- | --- |
| `static java.lang.String[]` | `createRandomWords(java.lang.String[] alphabet, int lengthOfWord, int numberOfWords)`             Creates pseudorandom Strings derived from an alphabet of String[] using the java.util.Random class. |
| `static java.lang.String` | `createRandowWord(int lengthOfWord)`             Returns a random word using nonambiguous alphabet. |
| `java.lang.String` | `getChallengeConcatinate()` |
| `java.lang.String` | `getDate()` |
| `java.lang.String` | `getHtmlChallengeTable()` |
| `static java.lang.String[]` | `getNonAmbiguousLetters()` |
| `static java.lang.String[]` | `getNumberLetters()` |
| `void` | `makeChallengeCard()` |
| `static void` | `makeHugeTable()`             An example in how to make a huge random word table. |

**Methods inherited from class java.lang.Object**

`equals, getClass, hashCode, notify, notifyAll, toString, wait, wait, wait`

**Field Detail**

### numberLetters

```
public static java.lang.String[] numberLetters
```

---


### nonAmbiguousLetters

```
public static java.lang.String[] nonAmbiguousLetters
```


**Constructor Detail**

### Passwords

```
public Passwords()
```


**Method Detail**

### makeChallengeCard

```
public void makeChallengeCard()
```

---


### createRandomWords

```
public static java.lang.String[] createRandomWords(java.lang.String[] alphabet,
                                                   int lengthOfWord,
                                                   int numberOfWords)
```

:   Creates pseudorandom Strings derived from an alphabet of String[] using the
    java.util.Random class. Indicate how long you want a particular word and
    the number of words.

---


### createRandowWord

```
public static java.lang.String createRandowWord(int lengthOfWord)
```

:   Returns a random word using nonambiguous alphabet. Don't use this method for creating more than one word!

---


### makeHugeTable

```
public static void makeHugeTable()
```

:   An example in how to make a huge random word table.

---


### getNonAmbiguousLetters

```
public static java.lang.String[] getNonAmbiguousLetters()
```

---


### getNumberLetters

```
public static java.lang.String[] getNumberLetters()
```

---


### getChallengeConcatinate

```
public java.lang.String getChallengeConcatinate()
```

---


### getDate

```
public java.lang.String getDate()
```

---


### getHtmlChallengeTable

```
public java.lang.String getHtmlChallengeTable()
```


---


|  |  |  |
| --- | --- | --- |
| **Overview** \| **Package** \| **Class** \| **Use** \| **Tree** \| **Deprecated** \| **Index** \| **Help** | |  |
| **PREV CLASS**   **NEXT CLASS** | **FRAMES**    **NO FRAMES**     **All Classes** |  |
| SUMMARY: NESTED \| FIELD \| CONSTR \| METHOD | DETAIL: FIELD \| CONSTR \| METHOD |  |


---
